# Description of Eight Skulls of Flathead Indians from Columbia River

**Published:** 1854-03

**Authors:** Carter P. Johnson

**Affiliations:** Professor of Anatomy and Physiology, Richmond, Va.


DESCRIPTION OF EIGHT SKULLS OF FLATHEAD INDIANS FROM COLUMBIA RIVER.—By CARTER P. JOHNSON, M. D., Professor of Anatomy and Physiology, Richmond, Va.—In the justly celebrated "Crania Americana" of Dr. Samuel George Morton, there are described the measurements of eight skulls of the Flathead tribes of Columbia river. In the eighth section of the second part of "Schoolcraft's history, condition, and prospect of the Indian tribes of the United States," upon the "Physical type of the American Indians," also written by Dr. Morton, (the last labor of his life) seven additional skulls of these tribes are described. These fifteen skulls are all of which I have seen any accurate measurements, though others may have been published in papers to which I have not had access. Any thing which may add to the slender stock of accurate statistical information which we possess with regard to these tribes will be interesting to the public and particularly to the medical men of America.

The skulls which I propose to describe were sent to me some time since by Dr. Charles H. Smith, U. S. A., and were taken by him from an Indian mound on the banks of the Columbia river in Oregon. Of the exact location of the mound he has not informed me, and I am therefore not able to state to which of the many Indian tribes inhabiting the banks of the Columbia river these skulls belonged.

The skulls are all in a good state of preservation, with one exception, they are all adult skulls, none however presenting indications of great age. They may therefore be taken as good types of the class to which they belong. The specimen No. 8 in the catalogue (vid. table), is not fully developed. The posterior molar teeth have not made their appearance at all, and one of the second molars has but just presented itself beyond the margin of the alveolar process; the remaining three second molars are fully out. In giving the mean of the measurements of these skulls this one will be omitted.

The appearance of these skulls is very singular, and with our idea of physical beauty, we find it difficult to credit the statement that such a conformation is actually regarded as a mark of beauty by those tribes among whom it is found, and that it is so highly valued that their slaves, who are for the most part derived from the adjacent tribes, are not allowed to imitate it. The most prominent deviations from the usual conformation of the skull are the great flattening of the frontal and occipital bones, and the consequent diminution of the antero-posterior diameter and the increase of the lateral diameter of the skull. The vertex is pushed backwards and the vertical diameter diminished. The basal aspect of the skull is very much altered; the face, retaining its usual width, seems very much disproportioned to the cranium, the lateral enlargement of which is here strikingly seen. These alterations exist of course in different degrees, in different specimens according to the degree and the duration of the compressing force employed, as will be evident from an examination of the table of measurements. Owing to the difficulty of applying the compressing force uniformly, I presume, very great distortion is sometimes produced. All of the skulls in my possession are strikingly nonsymmetrical, but two of them, Nos. 6 and 7 of the catalogue, are particularly distorted, and in both the flattening has been produced principally upon the left side, leaving the capacity of the right side of the cranium very much the greater. In two of the eight skulls which I have, the frontal suture remains entirely perfect from the sagittal to the fronto-nasal suture. Can this be in any manner the result of the compression of the frontal bones? In seven of the eight, " ossa wormiana " are found, some of them very large.

According to Dr. Morton,* this custom of flattening the head obtains among many tribes, among which are the Klickatats, Kalapooyahs, and Multnomahs of the Wallamet river and its vicinity; and the Chinouks, Clatsaps, Klatstonis, Cowalitsks, Kathlamets, Killemoohs and Chelakis of the lower Columbia and its vicinity. It may not be interesting to state the mode in which this curious deformity is produced by these numerous tribes. It will enable us to appreciate their moral sensibilities to some extent.

" The mode by which the flattening is effected," says Mr. Townsend, " varies considerably with the different tribes. The Wallamet Indians place the infant, soon after birth, upon a board, to the edges of which are attached little loops of hempen cord or leather, and other similar cords are passed in front and back, in a zigzag manner, through these loops, enclosing the child and binding it firmly down. To the upper edge of this board, in which is a depression to receive the back part of the head, another smaller one is attached by hinges of leather, and made to lie obliquely upon

* Crania Americana p. 203.

the forehead; the force of the pressure being regulated by several strings attached to its edge which are passed through holes in the board upon which the infant is lying, and secured there."*

"The mode of the Chinouks, and others near the sea, differs widely from that of the upper Indians, and appears somewhat less barbarous and cruel, a sort of cradle is formed by excavating a pine log to the depth of eight or ten inches. The child is placed in it on a bed of little grass mats and bound down in the manner above described. A little box of tightly plaited or woven grass is then applied to the forehead and secured by a cord to the loops at the side. The infant is thus suffered to remain from four to eight months or until the sutures of the skull have in some measure united, and the bone become solid and firm. It is seldom or never taken from the cradle, except in case of severe illness, until the flattening process is completed."

It is not wonderful that the result of this process, to which these unfortunate babies are subjected is "often ulceration of the scalp and perhaps, not unfrequently, death itself." Three of the skulls in my possession present distinct evidences of ulceration of the frontal bones themselves, and one of them, the youngest, presents upon the frontal bone and the adjacent part of the parietals, that honeycomb appearance so familiar to the pathologist as the result of syphilitic disease.

* Townsend, Journey to the Columbia River, p. 175.

